# Transient recurrent episodes of abducens nerve palsy and cheiro-oral syndrome in a sub-occlusive carotid bulb atherosclerotic stenosis

**DOI:** 10.1093/omcr/omae020

**Published:** 2024-03-25

**Authors:** Moussa Toudou-Daouda, Roger-Venant Yatwa-Zaniwe, Nana-Rahamatou Aminou-Tassiou, Nicolas Chausson, Didier Smadja

**Affiliations:** Department of Neurology, Centre Hospitalier Sud Francilien, Corbeil-Essonnes, France; Department of Neurology, Centre Hospitalier Sud Francilien, Corbeil-Essonnes, France; Department of Neurology, Centre Hospitalier Sud Francilien, Corbeil-Essonnes, France; Department of Neurology, Centre Hospitalier Sud Francilien, Corbeil-Essonnes, France; University of Paris, INSERM U1266, FHU NeuroVasc, France; Department of Neurology, Centre Hospitalier Sud Francilien, Corbeil-Essonnes, France; University of Paris, INSERM U1266, FHU NeuroVasc, France

**Keywords:** abducens nerve palsy, cheiro-oral syndrome, internal carotid artery, atherosclerotic stenosis, transient ischemic attacks

## Abstract

We report the case of a male in his 50s with a history of smoking admitted to our hospital for three transient recurrent episodes of less than 60 min of cheiro-oral paresthesias and binocular horizontal diplopia with convergent strabismus. On admission, his neurological examination was normal. Cerebral magnetic resonance imaging showed no cerebral lesion. Computed tomography angiography showed a sub-occlusive right carotid bulb atherosclerotic stenosis, the absence of abnormality of the subclavian arteries and the origin of the vertebral arteries, and no stenosis of the basilar artery or posterior cerebral arteries. Routine blood tests were normal with glycated hemoglobin of 6.5%. The patient underwent right carotid endarterectomy. One year after carotid endarterectomy, the patient has had no other cerebrovascular events.

## BACKGROUND

Extracranial internal carotid artery (ICA) atherosclerotic disease is one of the most common causes of anterior circulation ischemic strokes and transient ischemic attacks (TIAs). The usual transient visual symptoms related to the anterior circulation are transient visual field defect or transient monocular blindness [1]. Binocular diplopia (isolated or associated) related to extracranial ICA disease is rarely reported. To our knowledge, one case of binocular diplopia due to oculomotor nerve palsy related to extracranial ICA spontaneous occlusive dissection has been reported [2]. The present study reports the case of transient recurrent episodes of abducens nerve palsy (ANP) and cheiro-oral syndrome (COS) related to a sub-occlusive carotid bulb atherosclerotic stenosis.

## CASE REPORT

**Figure 1:**
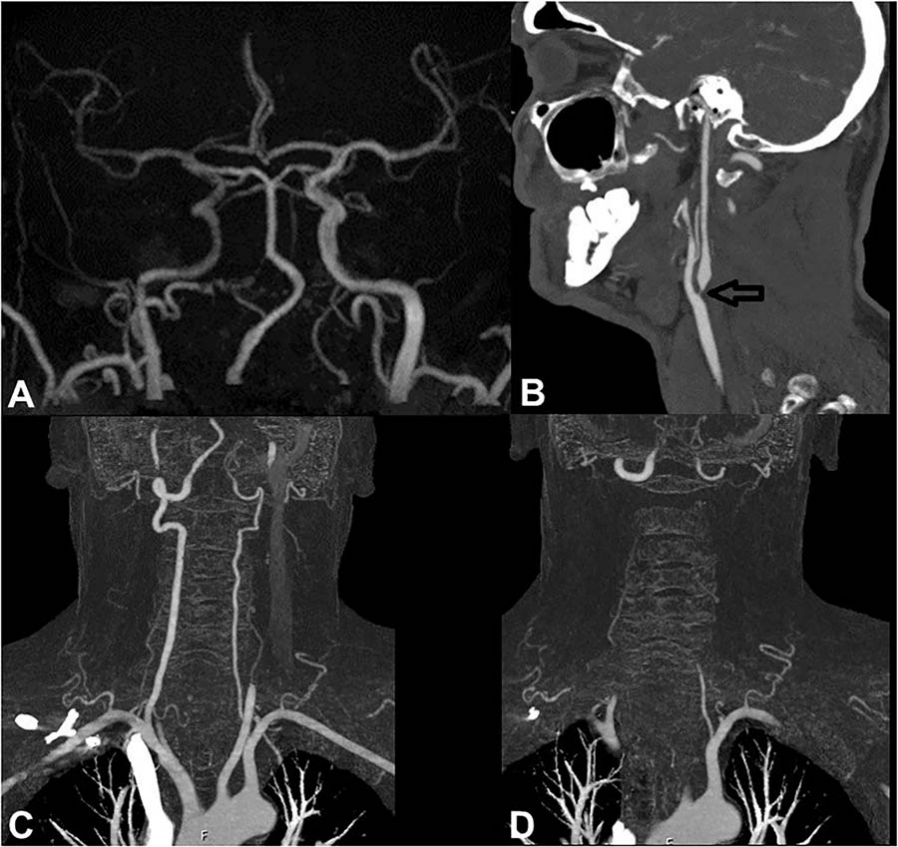
(**A**) Cerebral MRI showing on time of flight sequence showed a right intracranial ICA of small diameter and low-intensity signal compared to the left indicating a low blood flow. (**B–D**) CTA showing a sub-occlusive right carotid bulb atherosclerotic stenosis (black arrow), the absence of abnormality of the subclavian arteries and the origin of the vertebral arteries.

A 52-year-old Caucasian man with a past medical history of smoking presented to our hospital with three transient episodes of less than 60 min of left cheiro-oral paresthesias and binocular horizontal diplopia. All episodes occurred during activities of daily living. During one of the episodes, the patient’s wife observed convergent strabismus in her husband without precision on the side. On admission after the three neurological events, the neurological examination was normal. Blood pressure was 126/74 mm Hg and capillary blood glucose was 120 mg/l. Routine blood tests were normal with glycated hemoglobin of 6.5%. Cerebral magnetic resonance imaging (MRI) showed a right intracranial ICA of small diameter and low-intensity signal compared to the left, indicating a low blood flow without sub- and supratentorial parenchymal lesions (Fig. 1A). Computed tomography angiography (CTA) showed a sub-occlusive right carotid bulb atherosclerotic stenosis (Fig. 1B), the absence of abnormality of the subclavian arteries and the origin of the vertebral arteries (Fig. 1C and D) as well as the absence of stenosis of basilar artery or posterior cerebral arteries. The diagnosis of recurrent TIAs combining ANP and left COS related to a sub-occlusive right carotid bulb atherosclerotic stenosis was retained because the three episodes of neurological events occurred during activities of daily living, and no recurrence occurred when the patient was on strict bed rest. The patient underwent right carotid endarterectomy. One year after carotid endarterectomy, the patient has had no other cerebrovascular events.

## DISCUSSION

The present report describes a case of sub-occlusive right carotid bulb stenosis causing recurrent transient episodes of ANP and left COS without cerebral lesions. To our knowledge, this report is the first reported case of transient ANP due to carotid bulb atherosclerotic stenosis without cerebral lesions. Likely, the association of ocular motor nerve palsy with COS points mainly to the posterior circulation, particularly the pontine or thalamic involvement. However, cerebral MRI ruled out any brain involvement. The CTA also ruled out abnormalities of the subclavian arteries, the origin of the vertebral arteries, the basilar artery, and the posterior cerebral arteries. The absence of arterial abnormality in the posterior circulation supports the hypothesis of the probable relationship between sub-occlusive carotid bulb atherosclerotic stenosis and recurrent transient episodes of ANP and the COS. In addition, the three episodes of TIA occurred during activities of daily living, and no recurrences occurred when the patient was on strict bed rest.

**Figure 2:**
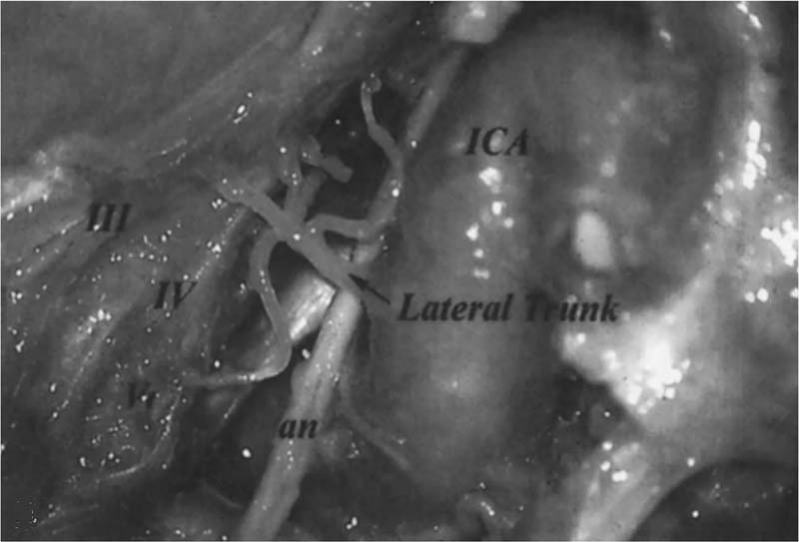
The branches of the lateral trunk of ICA which supply the oculomotor nerve (III), trochlear nerve (IV), abducens nerve (an), and ophthalmic nerve (V) on the lateral wall of the cavernous sinus (*Figure 3* from Tekdemir et al. [4]).

In their cisternal course, oculomotor, trochlear, and abducens nerves are supplied by branches of the basilar artery, the superior cerebellar artery, and the anterior inferior cerebellar artery, respectively [3]. However, in their intracavernous course, ocular motor nerves are supplied by the arterial trunks from the internal carotid artery, as shown in Fig. 2 [4]. The pathophysiological mechanism of the transient recurrent episodes of ANP in the present report would be hemodynamic, with low blood flow in the intracavernous portion of the ICA (as shown in Fig. 1A) causing hypoperfusion in the arterial trunks intended to vascularize the intracavernous segments of the ocular motor nerves, mainly abducens nerve. Among the extracranial ICA diseases, only one case report of spontaneous occlusive dissection causing isolated oculomotor nerve palsy has been reported [2]. The mechanisms mentioned by the authors in this clinical observation were embolism in the arterial trunk intended to vascularize oculomotor nerve, the hemodynamic mechanism (as suggested in our case report), or the combination of both mechanisms.

Regarding the transient recurrent episodes of the COS, their pathophysiological mechanism of occurrence is the same as that of ANP. The low blood flow in the intracavernous portion of the ICA causes hypoperfusion in the middle cerebral artery and, consequently, suffering ischemia mainly in the sensory cortex responsible for the COS. This syndrome has been previously reported in patients with postcentral gyrus involvement, called cortical COS [5].

In conclusion, the present case report showed the interest of searching for a stenotic disease of the extracranial ICA (such as dissection or atherosclerotic disease) in patients who consulted for acute permanent or transient ocular motor nerve palsy.

## Data Availability

All data generated or analyzed during this study are included in this published article.
